# Research on helmet wearing detection method based on deep learning

**DOI:** 10.1038/s41598-024-57433-z

**Published:** 2024-03-25

**Authors:** Lihong Wei, Panpan Liu, Haihui Ren, Dong Xiao

**Affiliations:** 1 School of Artificial Intelligence and Big Data, Hulunbeier University, Inner Mongolia, 021008 Hailar China; 2 Information Science and Engineering School, Northeastern University, Shenyang, 110004 China; 3 Liaoning Key Laboratory of Intelligent Diagnosis and Safety for Metallurgical Industry, Northeastern University, Shenyang, 110819 China

**Keywords:** Deep learning, Object detection, Helmet-wearing detection, YOLOv5, Attention mechanism, Engineering, Civil engineering, Electrical and electronic engineering, Mechanical engineering

## Abstract

The vigorous development of the construction industry has also brought unprecedented safety risks. The wearing of safety helmets at the construction site can effectively reduce casualties. As a result, this paper suggests employing a deep learning-based approach for the real-time detection of safety helmet usage among construction workers. Based on the selected YOLOv5s network through experiments, this paper analyzes its training results. Considering its poor detection effect on small objects and occluded objects. Therefore, multiple attention mechanisms are used to improve the YOLOv5s network, the feature pyramid network is improved into a BiFPN bidirectional feature pyramid network, and the post-processing method NMS is improved into Soft-NMS. Based on the above-improved method, the loss function is improved to enhance the convergence speed of the model and improve the detection speed. We propose a network model called BiFEL-YOLOv5s, which combines the BiFPN network and Focal-EIoU Loss to improve YOLOv5s. The average precision of the model is increased by 0.9% the recall rate is increased by 2.8%, and the detection speed of the model does not decrease too much. It is better suited for real-time safety helmet object detection, addressing the requirements of helmet detection across various work scenarios.

## Introduction

China is a big country in infrastructure construction. In the past 70 years, China has made remarkable achievements in infrastructure construction. The rapid development of the construction industry also drives the rapid development of the national economy. However, the construction site is very dangerous. According to statistics, the probability of accidents in the construction industry ranks first among all work industries^1,2^. Safety accidents can result in significant human and property losses. Therefore, while pursuing rapid economic development and vigorously implementing infrastructure projects, greater emphasis should be placed on the safety of construction workers’ lives. Among many construction safety accidents, the accidents of falling from high altitude account for more than half, and also include object impact accidents, collapse accidents, lifting equipment injury accidents, construction equipment injury accidents, and so on^3^. Wearing safety helmets is the most important means of preventing high fall accidents, which can greatly reduce casualties on account of construction safety accidents. As object detection and deep learning technology continue to advance, a multitude of scholars have begun to conduct research on object detection at construction sites^4,5^. However, because of the intricate conditions within the construction site, changeable weather and light, occlusion, dense personnel, and inconsistent object size, real-time detection on the site is difficult. At present, although there are many related studies, most of them can only be used for simple recognition in ideal environments, and it is difficult to carry out specific applications^6^.

As computer technology advances, the application of machine learning techniques has become increasingly prevalent in the detection of safety helmet usage. Currently, the detection algorithm for safety helmet usage can be categorized into two main types: the target detection algorithm that relies on traditional machine learning and the target detection algorithm that relies on deep learning. The conventional algorithm for detecting safety helmet usage primarily relies on recognizing color and shape features. In 2014, Liu et al.^7^ employed a hybrid approach that combined a Support Vector Machine (SVM) with skin tone detection to realize safety helmet recognition.In 2015, Shrestha et al.^8^ used Haar-like features to detect faces and used an edge detection algorithm to find helmet contour features. In 2016, Rubaiya et al.^9,10^ utilized the Histogram of Orientation Gradient (HOG) algorithm for detecting construction workers by combining frequency domain information in images. They further employed the Circle Hough Transform (CHT) feature extraction technique to determine whether the workers were wearing helmets. While helmet detection algorithms based on traditional machine learning, such as those mentioned above, have faster detection speeds, they require manual feature design and classifier training for specific detection objects. Additionally, due to the limited feature set and poor generalization ability, these algorithms cannot effectively detect targets in complex construction environments, leading to inaccurate detection results.

For the problem of helmet wear monitoring, research on deep learning-based target detection algorithms is the mainstream approach. Deep learning-based target detection algorithms can be categorized into two types: two-stage detection algorithms based on candidate frames and one-stage detection algorithms based on regression. Two-stage detection methods, such as RCNN^11^, Fast-RCNN^12^, and FasterRCNN^13^, use Region Proposal in the first stage to generate candidate regions and extract feature vectors from them. In the second stage, a convolutional neural network is utilized to forecast the category and position of the object, enabling accurate detection and localization of the target. On the other hand, single-stage-based detection methods, including YOLO^14^ and SSD^15^, do not require generating candidate regions. Instead, they directly input images and output the position and label of the entity, which significantly enhances the detection performance of the algorithm through the end-to-end technique. Compared to the two-stage target detection algorithm, the one-stage target detection algorithm achieves target location and classification results through a single forward inference from the network. As a result, the detection speed is significantly faster than the two-stage detection algorithm. In 2018, Fang et al.^16^ applied the Faster RCNN algorithm to helmet-wearing detection for the first time, and although the test accuracy was improved, it still could not meet the real-time demand. In 2021, Zhou et al.^17^ and 2022, Kisaezehra et al.^18^ used the YOLOv5 model for helmet detection to achieve high accuracy and speed to meet the real-time requirements, and the model generalization ability was poor due to the small sample of real scenes in the construction site of the dataset. In addition, some scholars have improved the classical target detection algorithm to achieve improved detection performance of the algorithm model. In 2022, Yang et al.^19^ made enhancements to the model backbone network based on YOLOv4. They utilized MCM modules with convolutional kernels of different sizes to boost the multi-scale feature extraction capability of the backbone network. Additionally, they incorporated the channel attention module to continuously concentrate on the characteristics of the channels for the detected tiny and indistinct objects. Finally, they replaced the CIOU loss function with EIOU to boost the model’s velocity of convergence and correctness of regression. In 2023, Chen et al.^20^ proposed to enhance the backbone network of YOLOv4. To boost the feature information, the lightweight network PP LCNet was utilized, and the coordinate attention mechanism module was integrated into the three output feature layers of the backbone network. Finally, they replaced the loss function with SIOU, which significantly reduced the model size while improving the detection speed after the enhancement. In 2023, a new super-resolution reconstruction module was designed by Han et al.^21^ for high-speed detection of high-resolution helmets. The module utilized a multi-channel attention mechanism to enhance the range of feature recognition. Additionally, a new CSP (cross-stage partial) module was proposed to alleviate information loss and gradient confusion.

Although the above method has optimized and improved the algorithm, it also has shortcomings in detecting small and dense targets, for the shortcomings, shortcomings of the existing technology, this paper proposes an improved helmet-wearing detection model BiFEL-YOLOv5s. Firstly, to effectively solve the problem of detecting objects with obvious size differences, the feature pyramid network is improved by using a weighted bi-directional feature pyramid network Bi-FPN instead of PANet, which introduces the concept of weights to improve the model performance. Secondly, the SENet attention mechanism is introduced to enhance the attention to the detection target, and comparative experiments are conducted in the dataset to verify its effectiveness. The Focal-EIoU Loss loss function is also used instead of the CIoU Loss loss function to improve the convergence of the model. Improved post-processing method using Soft-NMS to address the presence of occlusion in helmet targets. The improved algorithm, compared with the traditional detection methods, improves the precision and recall while also effectively improving the problem in scenarios with dense targets, small targets, and occluded targets, and meets the performance requirements for helmet wear detection at construction sites.

## Proposed methods

The YOLO family of methods is today’s most popular single-stage detection technique, compared with other two-stage target detection algorithms, they do not need to generate a candidate region, but rather, the image will be input from the input side, and then in the output side of the output of the target’s location and category, this end-to-end technology greatly improves the detection performance of the algorithm. Meanwhile, with the continuous development of the YOLO series of algorithms, several algorithms from YOLOv1 to v7 have been produced, and the performance has been continuously improved. Among them, YOLOv5^22^ is currently the most widely used, with high accuracy, speed, comprehensiveness, and many other advantages. Compared to YOLOv6 and YOLOv7, the YOLOv5 model is the smallest, consumes the least amount of resources, is the fastest in training and inference, and only slightly inferior in detection accuracy. At the same time, due to the limited performance of most of the equipment applicable in the construction environment and the small budget, it is necessary to choose the appropriate detection model, i.e., a smaller number of parameters, a smaller amount of computation and moderate accuracy, and to ensure that it can be used in some simple application scenarios, so in this paper, we choose the YOLOv5 model through comprehensive consideration.

### Loss function

Within the realm of object detection, it is customary to employ Intersection over Union (IoU) for assessing the discrepancy between the predicted bounding box and the ground truth box during loss function computation. IoU is defined as the ratio of intersection and union of two bounding boxes. The detection box is denoted as A, and the true box is denoted as B. Then IoULoss is the negative logarithm of IoU, often written as $IoULoss = 1 - IoU = 1 - \left( {A \cap B} \right)/\left( {A \cup B} \right)$. Compared with L1/L2 Loss using 4 coordinate points for regression, IoU Loss is scale-invariant and its output value is between (0,1), so it can better reflect the deviation between the predicted box and the ground truth.

The bounding box loss of YOLOv5 uses CIoU loss, although the overlapping area of the predicted box and the real box, the distance from the center point, and the aspect ratio are taken into account, the parameter v in the formula representing the consistency of the aspect ratio indicates only the difference in the horizontal and vertical ratios and does not take into account the width and the height separately. Therefore, if the predicted frame has exactly the same aspect ratio as the real frame, it will also cause its penalty term to remain at 0. The gradient of w、h with respect to v in CIoU loss is a pair of opposites, w and h cannot increase or decrease at the same time.YOLOv5 also uses CIoU Loss as the loss function, and its formula is shown in Eq. (1):

1 $$ \begin{array}{*{20}c} {L_{CIoU} = 1 - CIoU = 1 - IoU + \frac{{\rho^{2} \left( {b,b^{gt} } \right)}}{{c^{2} }} + \frac{{v^{2} }}{{\left( {1 - IoU} \right) + v}}} \\ \end{array} $$

where *v* is a parameter representing the consistency of aspect ratio as shown in Eq. (2):

2 $$ \begin{array}{*{20}c} {v = \frac{4}{{\pi^{2} }}\left( {arctan\frac{{w^{gt} }}{{h^{gt} }} - arctan\frac{w}{h}} \right)^{2} } \\ \end{array} $$

While CIoU Loss considers aspects such as the overlap area, center point distance, aspect ratio, and other factors, the parameter “v” in the formula only signifies the dissimilarity in aspect ratio, without individual consideration of width and height. Therefore, EIoU Loss is proposed to consider the aspect ratio separately. In addition, Focal Loss is added to make the regression process focus on high-quality anchors, which solves the problem of loss value oscillation caused by low-quality samples. The Loss function of EIoU Loss is mainly composed of three parts, IoU loss $L_{IoU}$, center distance loss $L_{dis}$, and width and height loss $L_{asp}$. In the calculation of width and height loss, the side length is directly used as the penalty term. The formula of EIoU Loss is shown in Eq. (3):

3 $$ \begin{aligned} L_{EIoU} & = L_{IoU} + L_{dis} + L_{asp} \\ & = 1 - IoU + \frac{{\rho^{2} \left( {b,b^{gt} } \right)}}{{c^{2} }} + \frac{{\rho^{2} \left( {w,w^{gt} } \right)}}{{C_{w}^{2} }} + \frac{{\rho^{2} \left( {h,h^{gt} } \right)}}{{C_{h}^{2} }} \\ \end{aligned} $$

Here, $C_{w}$ and $C_{h}$ are the width and height of the minimum bounding rectangle of the two bounding boxes, respectively. On the basis of EIoU Loss, the authors introduce Focal Loss, whose penalty formula is shown in Eq. (4):

4 $$ \begin{array}{*{20}c} {L_{Focal - EIoU} = IoU^{\gamma } L_{EIoU} } \\ \end{array} $$

The CIoU Loss function is employed as the bounding box loss in the YOLOv5 model. However, considering that CIoU Loss is only a regression on aspect ratio, in this paper, the Focal-EIoU Loss function is implemented as the bounding box loss in YOLOv5 predictions to enhance its performance. Not only the length and width are considered separately, directly using the side length for regression will further accelerate the regression process, but also the added Focal Loss model can optimize the sample imbalance problem in the bounding box regression task, further improving the performance of the model.

### Attention mechanism

The primary function of the attention mechanism is to determine which portions of the input require focused consideration, allocate resources judiciously, and allocate greater resources to the critical components. In the domain of object detection, the introduction of an attention mechanism notably amplifies the detection performance for small objects. Therefore, this paper compares the effect of three attention mechanisms for safety helmet object detection. The three attention mechanisms are SeNet, CBAM, and CA attention mechanisms. These three attention mechanisms were chosen because they are typical of channel domains and hybrid domains. The attention mechanism is analyzed according to the model structure and is divided into three major attention domains: (1) channel domain: similar to the signal on each channel added a weight to represent the channel with the key information of the relevance of the words, the larger this weight, the higher the relevance is indicated. A mask mask is generated for the channel, scored, and represented as SeNet. (2) Hybrid domain: the attention of the spatial domain is to ignore the information in the channel domain and treat the picture features in each channel equally, this practice will limit the spatial domain transformation method to the original picture feature extraction stage, and the interpretability of the application to other layers of the neural network layer is not strong. The attention of the channel domain is a direct global average pooling of the information in a channel while ignoring the local information in each channel, this practice is actually also a more violent behavior. So by combining the two ideas, a hybrid domain model of the attention mechanism can be designed. Simultaneously evaluating and scoring channel attention and spatial attention is represented by CBAM.

#### SeNet attention module

**Figure 1:**
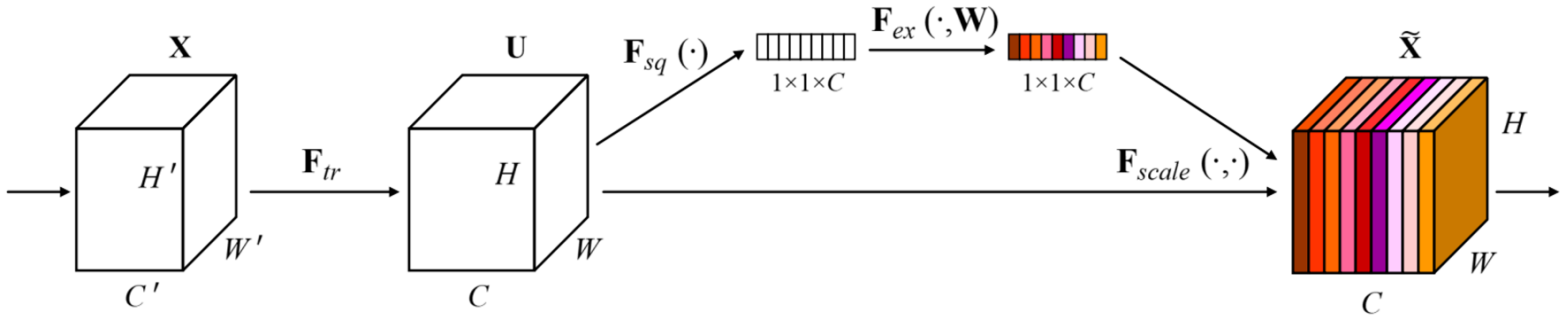
The structure of SeNet module.

SeNet (Squeeze-and-Excitation Networks) attention module^23^ was proposed by Hu et al., in 2017, and its network structure is shown in Fig. 1^23^. SeNet is categorized as a channel attention mechanism, prioritizing the inter-channel relationships for weight learning among different channels. The SeNet module comprises three components: the squeeze operation, excitation operation, and scale operation.

The feature map is compressed using the global average pooling technique. The size of the feature map is compressed from $H \times W \times C$ to $1 \times 1 \times C$, where *C* represents the number of channels of the input feature map. The channel global spatial feature is transformed into a global feature by squeeze operation. In excitation operation, two fully connected layers are used to realize the transformation of the size of the feature map from $1 \times 1 \times C$ to $1 \times 1 \times C^{*}$ and then to $1 \times 1 \times C$, where $C^{*} = C/16$ can achieve a balance between computational complexity and performance. The initial fully connected layer decreases the dimension of the feature map and feeds it into the ReLU function to apply nonlinear activation. The second fully connected layer elevates the dimensionality of the feature map and directs it into the sigmoid function to constrain the output values within the range of 0 to 1. The excitation operation aims to recalibrate features, reduce the complexity of model parameters, and enhance the generalization ability of the model. In the scale operation, the weight parameters of the output $1 \times 1 \times C$ in the excitation operation are multiplied by the corresponding channels with the input $H \times W \times C$ feature map to achieve the assignment of weights.

#### CBAM attention module

**Figure 2:**
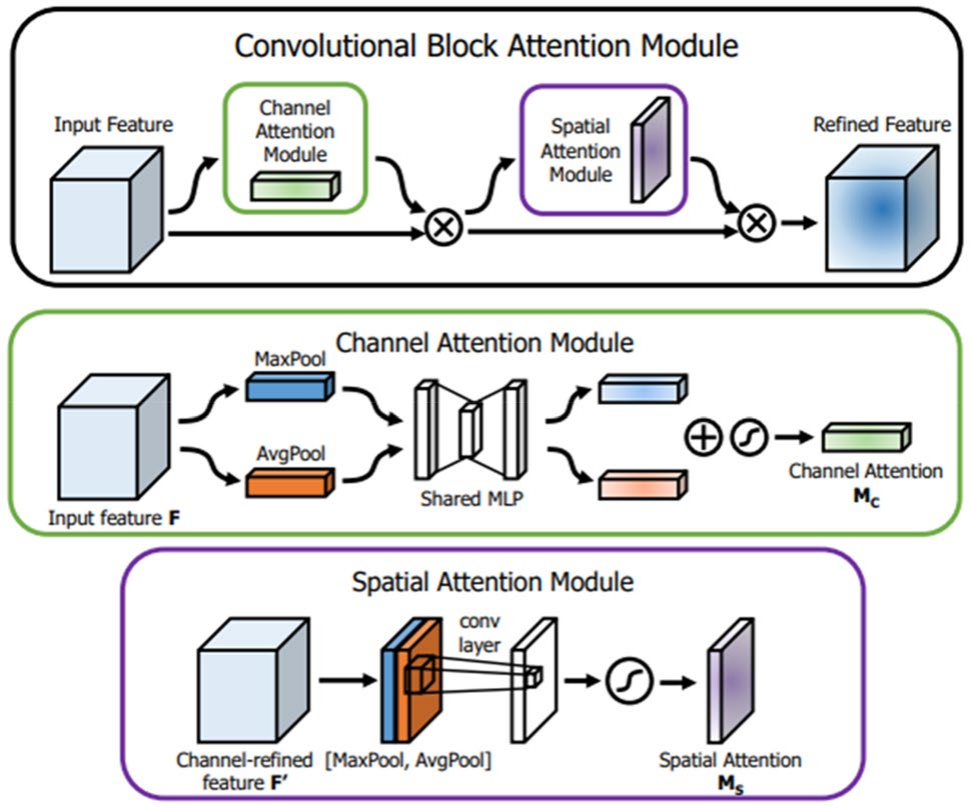
The structure of CBAM module.

CBAM (Convolutional Block Attention Module) attention module^24^ is an attention mechanism that integrates channel attention and spatial attention proposed by Woo et al., in 2018, which is an extension of SeNet attention. The network’s capability for feature extraction is further enhanced, and its structural diagram is depicted in Fig. 2^24^.

The CBAM attention module comprises two components: the channel attention module and the spatial attention module, which assign weights to the channel layer and spatial layer, respectively. In the first part, the channel attention module is similar to the SeNet attention module. However, the global Max pooling and global average pooling are respectively applied to the $H \times W \times C$ dimensional feature maps in CBAM to obtain two $1 \times 1 \times C$ dimensional feature maps, which are still input into the two fully connected layers for dimension reduction and dimension enhancement. The two acquired feature maps are summed and fed into the sigmoid activation function, after which the final weight parameter is applied to the input feature map through multiplication. The inclusion of global average pooling encourages the neural network to prioritize global information within the image and enhance the semantic content of the primary channel.

In the second part, spatial attention also inputs the $H \times W \times C$ dimension feature maps into the global Max pooling and global average pooling to obtain two $H \times W \times 1$ feature maps, concatenate the two feature maps, and input the obtained $H \times W \times 2$ feature maps into a convolution layer to obtain $H \times W \times 1$ feature maps. It is fed into the sigmoid function to derive a weight parameter with an output value ranging between 0 and 1, and this parameter is then multiplied with the feature map output from the channel attention mechanism to obtain the ultimate attention feature. CBAM can be embedded into the residual network and combined with the conv module or C3 module in YOLOv5 for improvement.

#### CA Attention module

**Figure 3:**
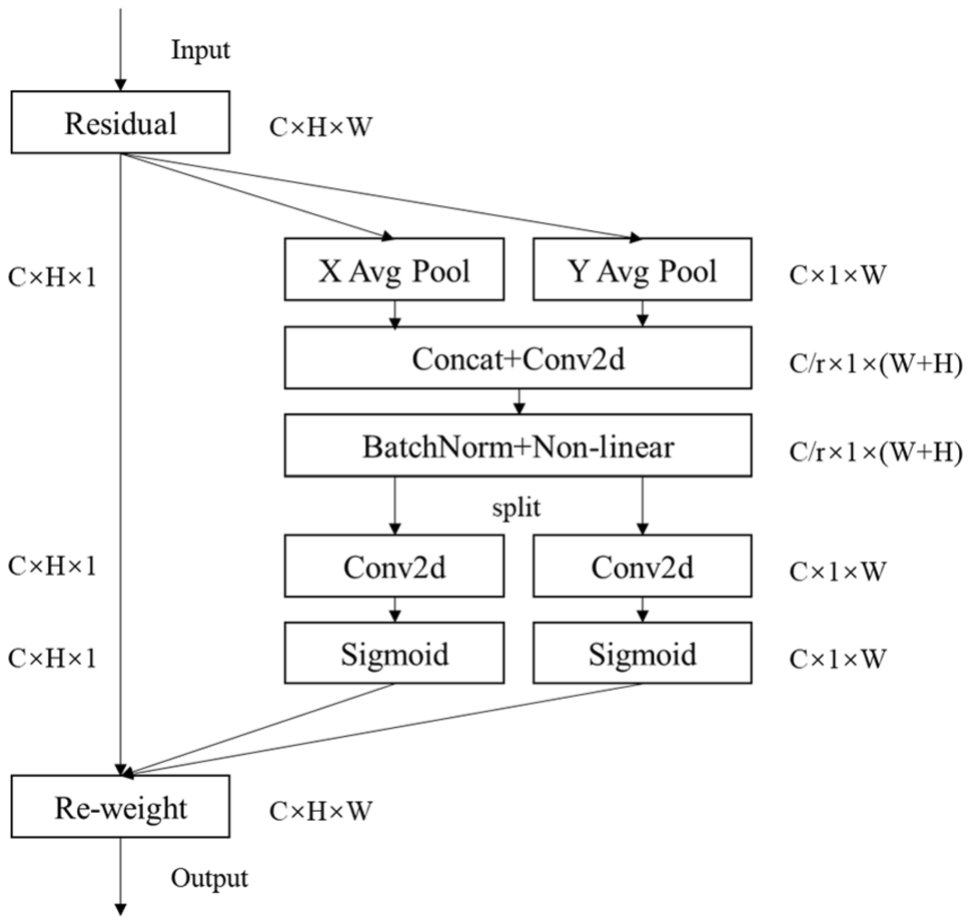
The structure of CA module.

CA (Coordinate Attention) attention module^25^ is an attention module proposed by Hou et al., which considers channel relationship and location information simultaneously. It is an improved strategy based on SeNet module, and its structural diagram is illustrated in Fig. 3^25^. By encoding the lateral and vertical position information into channel attention, we can focus on a wide range of position information while only adding a small amount of computation.

The CA module consists of two stages: coordinate information embedding and coordinate attention generation. In the CA module, two pooling kernels with dimensions of $\left( {{\text{H}},1} \right)$ and $\left( {1,{\text{ W}}} \right)$ are used to channel encode the input feature maps along the vertical and horizontal coordinates, respectively, to obtain a pair of direction-aware feature maps, which can obtain accurate position information and enhance the positioning accuracy of the network. The output of the CTH channel at the height “h” and width “w” is represented in Eqs. (5) and (6), correspondingly.

5 $$ \begin{array}{*{20}c} {z_{c}^{h} \left( h \right) = \frac{1}{W}\mathop \sum \limits_{0 \le i < W} x_{c} \left( {h,i} \right)} \\ \end{array} $$

6 $$ \begin{array}{*{20}c} {z_{c}^{w} \left( w \right) = \frac{1}{H}\mathop \sum \limits_{0 \le j < H} x_{c} \left( {j,w} \right)} \\ \end{array} $$

During the coordinate attention generation phase, the coordinate information is embedded into the results in the module for concatenate operation and 1 × 1 convolution transformation operation. Among them, the concatenate operation performs encoding operations along both horizontal and vertical directions to generate intermediate feature maps for spatial information encoding. To mitigate the model’s complexity, the number of channels is reduced to 1/32 of the original, the intermediate feature map is divided into two tensors along the spatial dimension, and the 1 × 1 convolution operation is used again. The output result obtained is used as the attention weight, and the horizontal and vertical weights are added to the input feature map at the same time in the way of the product.

#### Comparison of three attention mechanisms

The SE attention mechanism enhances the useful information in the feature map by learning the importance of global channels. Its advantage is that it is computationally simple and can efficiently extract global features, but its disadvantage is that it does not consider spatial correlation.

The advantage of CA attention mechanism is that not only channel information but also direction-related position information is considered. The disadvantage is that it requires additional computation with high computational overhead. In addition, it is not possible to capture long-range dependencies because it requires the computation of attention weights for the whole feature map.

The advantage of CBAM attention mechanism is that it introduces two analysis dimensions, spatial attention and channel attention, and realizes the sequential attention structure from channel to space. The disadvantage is that it requires more computational resources and higher computational.

### Non-maximum suppression

In object detection, there may be multiple prediction boxes for the same detection object. In order to retain the optimal prediction box, the Non-Maximum Suppression (NMS) technique is applied during post-processing to eliminate redundant bounding boxes and yield the ultimate detection outcome. The main process of NMS is to first sort all the predicted boxes according to their confidence scores, find the bounding box with the highest score, calculate the overlap degree (IoU value) between the remaining bounding boxes and the highest scoring bounding boxes, eliminate the bounding boxes that are greater than the threshold, and then find the highest confidence score in the remaining bounding boxes, and continue the above process until all the remaining bounding boxes are eliminated. This NMS is adopted as a post-processing method in YOLOv5.

However, the NMS method, on the one hand, is difficult to determine the threshold of IoU, on the other hand, it will produce false suppression for different objects with large overlap. Therefore, this paper proposes to use Soft-NMS to improve the YOLOv5s model. In the improved method, Soft-NMS is applied to the inference stage, while NMS is still used for computation in the training stage. The Soft-NMS method mainly improves the problem of false deletion of overlapping objects in NMS. In the NMS algorithm, the detection boxes whose IoU value with the detection box M is greater than or equal to the threshold $N_{t}$ will be deleted directly, so the corresponding false deletion problem will be caused. For the NMS method, the formula for calculating the confidence score is shown in Eq. (7):

7 $$ \begin{array}{*{20}c} {s_{i} = \left\{ {\begin{array}{*{20}c} {s_{i} } \\ 0 \\ \end{array} { }} \right.\begin{array}{*{20}c} { IoU\left( {M,b_{i} } \right) < N_{t} } \\ { IoU\left( {M,b_{i} } \right) \ge N_{t} } \\ \end{array} } \\ \end{array} $$

The improvement of Soft-NMS is that the overlapping detection boxes are not deleted directly, but their confidence scores are reduced, and the corresponding strategy is designed to ensure that the overlapping object detection boxes are retained while multiple detection boxes of the same object are deleted. The confidence score of Soft-NMS is calculated as shown in Eq. (8):

8 $$ \begin{array}{*{20}c} {s_{i} = \left\{ {\begin{array}{*{20}c} {s_{i} } \\ {s_{i} \left( {1 - IoU\left( {M,b_{i} } \right)} \right)} \\ \end{array} \begin{array}{*{20}c} {} & {IoU\left( {M,b_{i} } \right) < N_{t} } \\ {} & {IoU\left( {M,b_{i} } \right) \ge N_{t} } \\ \end{array} } \right.} \\ \end{array} $$

The calculation formula of Gaussian function $s_{i}$ is shown in Eq. (9):

9 $$ \begin{array}{*{20}c} {s_{i} = s_{i} e^{{ - \frac{{tan\left( {M,b_{i} } \right)^{2} }}{\sigma }}} ,\forall b_{i} \notin D} \\ \end{array} $$

### Improved feature pyramid networks

**Figure 4:**
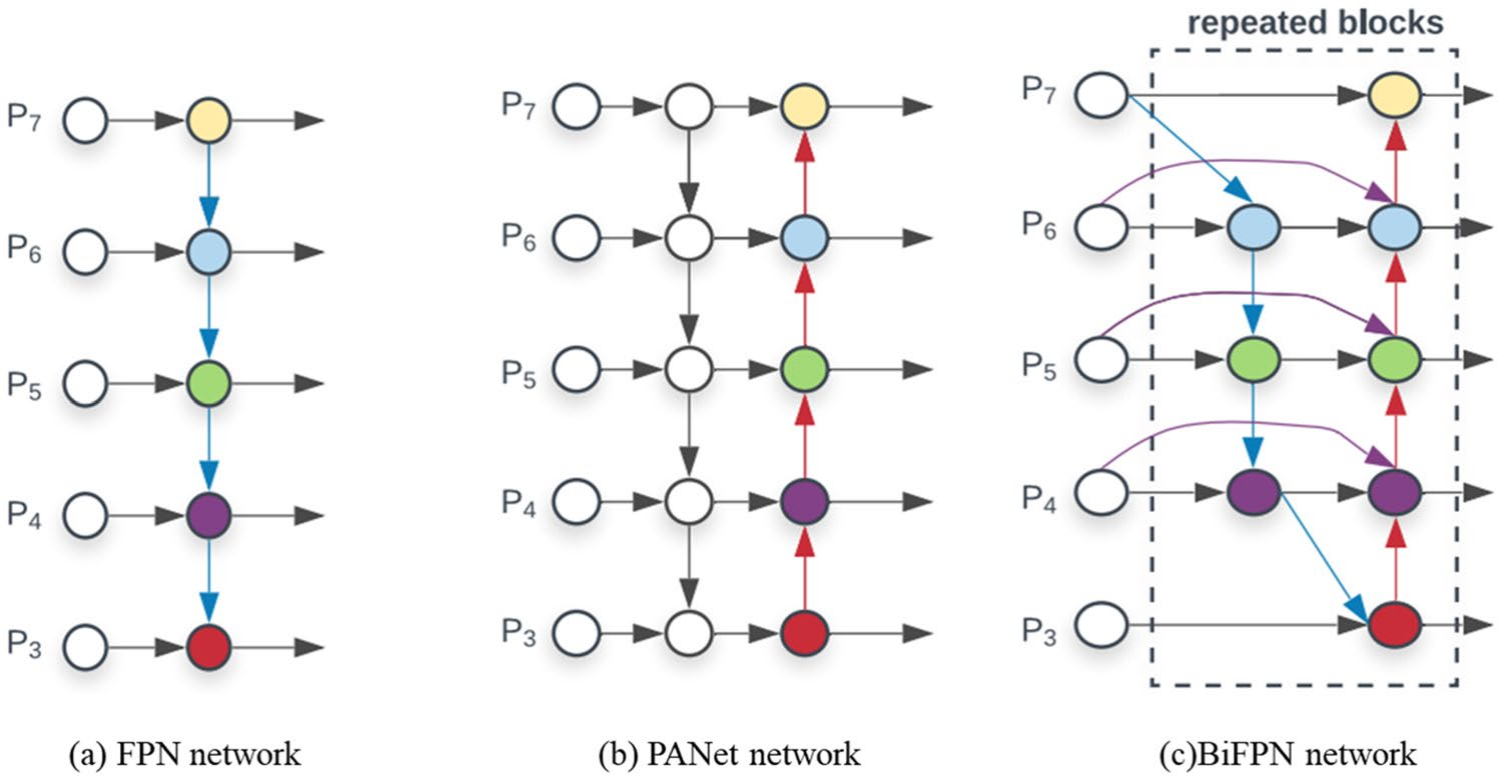
Network structure diagram of the three networks FPN, PANet, and BiFPN.

YOLOv5s Network adopts the structure of FPN + PANet in the Neck part. In order to further improve the efficiency of the multi-scale fusion of the model, this paper intends to use BiFPN (Bidirectional Feature Pyramid Network)^26^ to improve the neck network of YOLOv5, and its structure is shown in Fig. 4a^26^. In BiFPN, considering that there is no feature fusion, the node with only one input edge will have little contribution to the feature network fusion of different features. Therefore, on the basis of the PANet network, as shown in Fig. 4b^26^, the intermediate nodes of P3 and P7 are removed to obtain a simplified feature fusion network. Jump connections are added to the input and output nodes of the same scale of the simplified network, as the purple arrows in Fig. 4c^26^, to fuse more features. Finally, for feature fusion of different resolutions, the previous network treats the input features with the same weight, but in fact, the contribution of features of different resolutions to the output features is not equal. Therefore, this paper proposes to increase the weight value of each input feature for feature fusion, so that the network learns the importance of different input features. Using Fast normalized fusion, the weight values are normalized and scaled to between [0, 1], similar to the Softmax method but with higher training speed.

### Statement of informed consent

The helmet images used in the manuscript were reportedly taken from the open-source helmet dataset SHWD, and all human participants involved in the images agreed to participate in the study and also agreed to the publication of identifiable information/images in an online open access publication.

## Dataset and results

### Selection and description of the dataset

**Figure 5:**
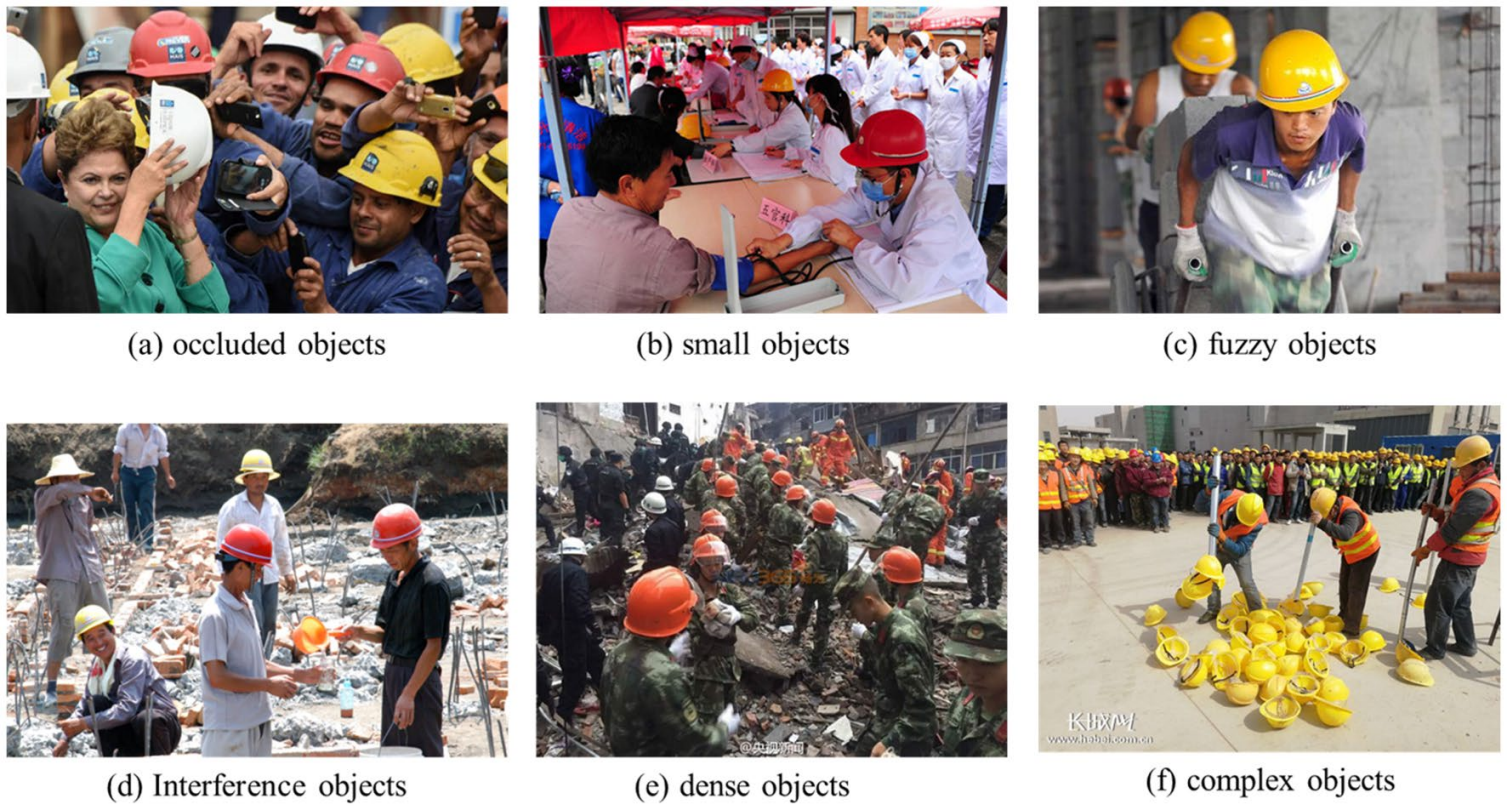
Different kinds of samples in public datasets.

In this study, an open-source safety helmet dataset SHWD (Safe Helmet Wearing Dataset, https://github.com/njvisionpower/Safety-Helmet-Wearing-Dataset) available on the internet, comprising a total of 3241 images, is utilized. The data set contains a certain number of positive and negative object samples of various types of safety helmets, such as small objects, objects with occlusion, dense objects, fuzzy objects, and interference objects wearing other hats, as shown in Fig. 5.

**Figure 6:**
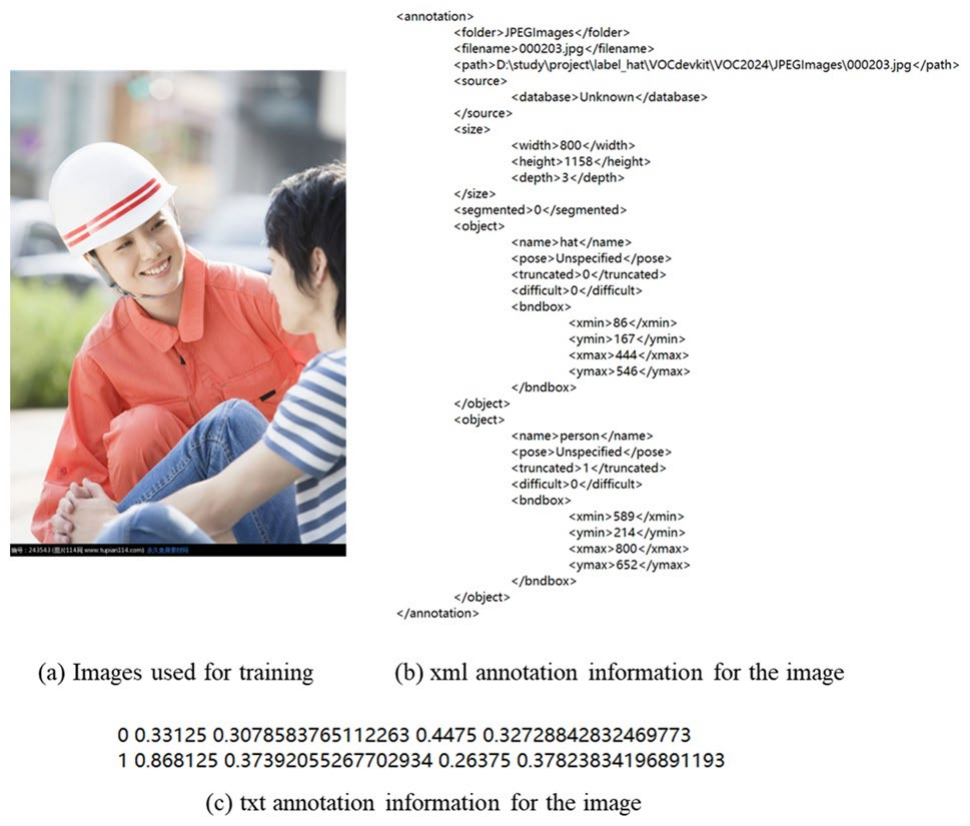
Safety helmet target detection image and its corresponding xml, txt annotation information.

The dataset contains two kinds of labels, hat, and person, indicating the staff wearing safety helmets and the staff not wearing safety helmets. In the helmet dataset used in this paper, the training set accounts for 80% of the total dataset, and the validation and test sets each account for 10%. The labeling method of the picture is consistent with the previous labeling method. The previous labeling method is for the helmet picture labeling is generally divided into two categories, one is wearing a helmet personnel, with “hat” labeling, the helmet and the head as a whole to labeling, and the other is not wearing a helmet personnel, with “person” labeling, construction personnel head labeling. The label file of the picture is in xml format, and its category and border position can be easily read out. As shown in Fig. 6, Fig. 6a is the object detection picture of the safety helmet, Fig. 6b is the tag information of its xml file, which can be read from the figure, the picture is an RGB image, the size is 800 × 1158, the picture contains two bounding boxes, hat, and person, where the coordinate of hat is (86, 167, 444, 546), and the coordinate of hat is (86, 167, 444, 546). Person’s coordinate is (589, 214, 800, 625). Read the information in the xml file, mark hat and person as 0 and 1 respectively, calculate the center point coordinates of the bounding box and its width and height value and normalize them. Each line represents an object, which is the category, the center point coordinates, and the width and height value, as shown in Fig. 6c, thus converting the xml file into a txt file suitable for the YOLO model.

### Experimental environment

In the experimental stage, this paper conducts experiments on the selection of the basic network, the selection of the attention mechanism, and the overall improvement effect of the network, respectively, and describes the results and the reasons for the selection in detail. The hardware configuration used in the experiment is the CPU model of 11th Gen Intel(R) Core(TM) i7-11800H, and the refresh frequency is 2.30GHz. The GPU version is NVIDIA GeForce RTX 3050 Ti Laptop GPU with 4GB of memory. The main hard disk model is Micron MTFDKBA512TFH with a capacity of 512G. The main board model is LNVNB161216. In this paper, the Pytorch deep learning framework is used when training the safety helmet object detection model. The running environment is Windows 10, the 64-bit operating system, the python version number is 3.9, the pytorch version number is 1.12.1, and the CUDA version number is 11.3.

During model training, the pre-trained weights trained by the public dataset MS COCO are used, which contains more than 330K images, 1.5 million objects, and 80 categories. The pre-trained outcomes serve as initialization parameters in the training process, followed by fine-tuning the parameters using the safety hat dataset introduced in section “Attention mechanism” of this paper. The configuration of the model's fundamental parameters is outlined in Table 1.

**Table 1:** The basic parameters of model.

Parameters	Value
Batch size	8
Epoch	100
Input size	640 × 640
Learning rate	0.01
Momentum	0.937
Weight decay	0.0005
IoU threshold (NMS)	0.6
confidence threshold	0.001

### Evaluation index

Throughout the experimental procedure, Precision, Recall, Average Precision (AP), and mean Average Precision (mAP) are chosen as the assessment metrics for detection accuracy, while Frames Per Second (FPS) is used as the speed evaluation criterion.

If the intersection ratio between the predicted outcome and the True value exceeds a specific threshold, the prediction is considered True. Employing this approach, the predicted results can be categorized into four scenarios: True Positive (TP), False Positive (FP), True Negative (TN), and False Negative (FN).

Among them, accuracy refers to the probability of correct detection among all detected objects, so its formula is expressed as Eq. (10):

10 $$ \begin{array}{*{20}c} {Precison = \frac{TP}{{TP + FP}}} \\ \end{array} $$

Recall refers to the probability of correct detection among all positive samples, so its formula is expressed as Eq. (11):

11 $$ \begin{array}{*{20}c} {Recall = \frac{TP}{{TP + FN}}} \\ \end{array} $$

The Precision-Recall curve (PR curve) is a graphical representation that plots Recall (R) on the horizontal axis and Precision (P) on the vertical axis. Average Precision (AP) represents the model accuracy under different recall rates, which refers to the area enclosed by the PR curve and the horizontal axis on the graph. Therefore, for a continuous PR curve, the formula is expressed as Eq. (12):

12 $$ \begin{array}{*{20}c} {AP = \mathop \smallint \limits_{0}^{1} {\text{PRdr}}} \\ \end{array} $$

Mean Average Precision (mAP) is the average of the precision values associated with various object categories, with its formula defined as depicted in Eq. (13). In this paper, n equals 2, representing hat and person, respectively.

13 $$ \begin{array}{*{20}c} {mAP = \frac{{\mathop \sum \nolimits_{{{\text{i}} = 1}}^{{\text{n}}} {\text{AP}}_{{\text{i}}} }}{{\text{n}}}} \\ \end{array} $$

Frames per second (FPS) represents the quantity of images processed in a single second, serving as a metric for evaluating the speed of object detection. When evaluating this metric, you need to ensure that the test environment is consistent. It can not only compare the number of images processed per unit of time, the unit is one, the larger the faster the detection speed, but also compare the detection time required to process a single image, the general unit is ms, the smaller is the faster detection speed.

### Results

#### Experiments on the selection of the base network

**Figure 7:**
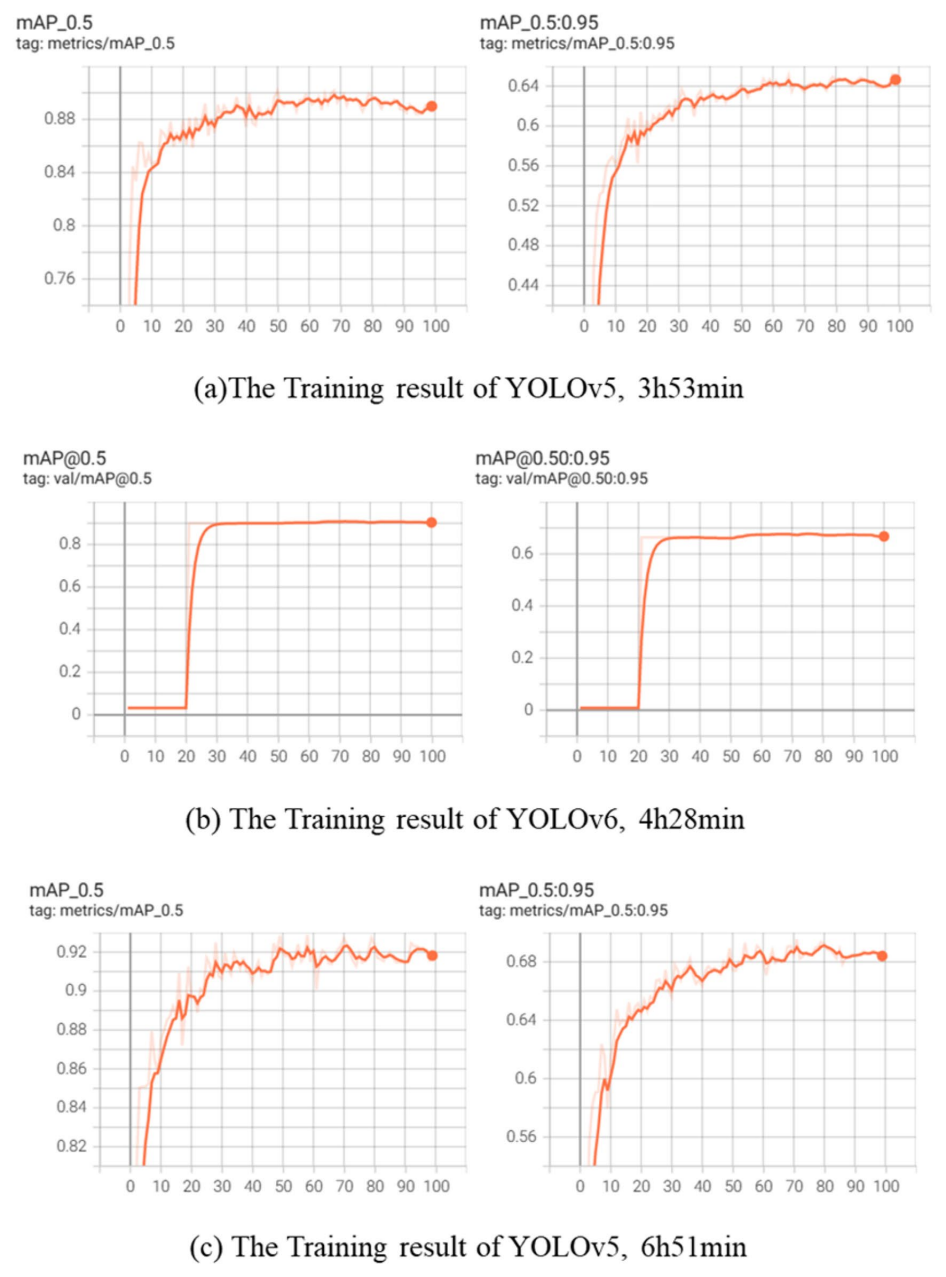
Training results and training time using YOLOv5-v7 respectively under the same conditions.

The data set mentioned in 2.2 of this paper is used to train and test YOLOv5, YOLOv6, and YOLOv7, and the training results and training time are shown in Fig. 7, where (a), (b), and (c) denote the change of average accuracy of YOLOv5, YOLOv6, and YOLOv7 in the training process with IoU of 0.5 and 0.5: 0.95 respectively.

The trained model undergoes testing on the identical test dataset to compare the average accuracy and detection speed among the three models, with the results presented in Table 2.

**Table 2:** Test results of YOLOv5-v7 algorithm.

Algorithm	mAP(IoU = 0.5)	mAP(IoU = 0.5:0.95)	FPS
YOLOv5	0.819	0.573	96.2
YOLOv6	0.838	0.604	68.4
YOLOv7	0.876	0.629	52.6

It can be seen from the experimental results that the average accuracy of YOLOv5 network is 81.9%, and the frame per second rate is 96.2, which achieves a satisfactory accuracy under the condition of high detection speed and less training time. The detection speed of YOLOv6 network is greatly reduced when the average accuracy is not improved. Although the average accuracy of YOLOv7 network is higher than that of YOLOv5, its detection speed is almost halved, and the training time of YOLOv7 network is twice that of YOLOv5 network. More importantly, its hardware equipment, such as video memory and other conditions, is not suitable for a wide range of popularity in the industrial safety helmet detection scene. Therefore, this paper chooses YOLOv5s network as the basic network for further research and improvement.

#### Selection of attention mechanism

**Figure 8:**
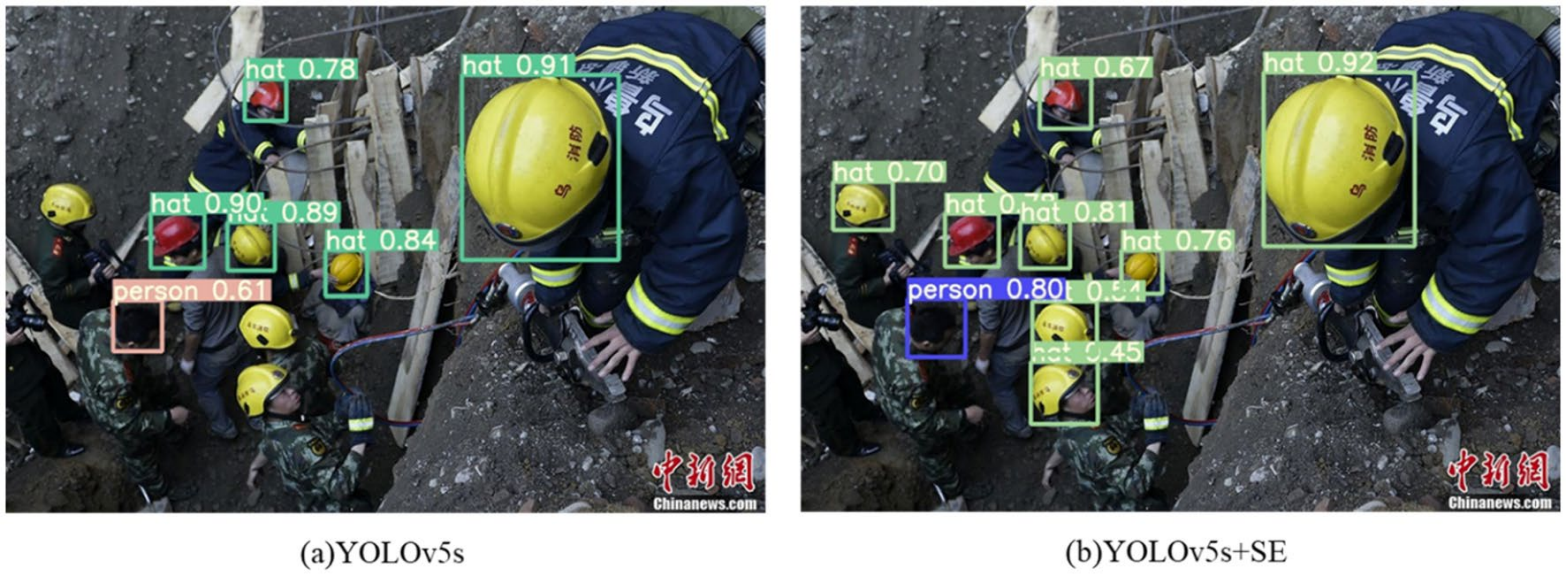
Detection results before and after adding SE attention mechanism.

Regarding the enhancement of the foundational network, this paper first considers the shortcomings of YOLOv5s network for small object detection, so three different attention mechanisms SE, CBAM, and CA are added to the backbone of YOLOv5s network, and the detection effects of the three attention mechanisms are compared. The test outcomes are displayed in Table 3. The CA attention mechanism will slightly improve the accuracy but cause a large decrease in the average precision. The CBAM attention mechanism does not result in a discernible enhancement in the overall accuracy of the network. The SE attention mechanism achieves an average accuracy increase of 0.9% while the detection speed and accuracy are almost unchanged. Therefore, it is finally decided to choose the SE attention mechanism to combine with other methods for further improvement. Figure 8 illustrates the contrast between detection results before and after the inclusion of the SE attention mechanism.

**Table 3:** Experimental results of adding attention mechanism.

Attention mechanism	mAP (IoU = 0.5)	mAP (IoU = 0.5:0.95)	P	R	FPS
YOLOv5s	0.819	0.573	0.87	0.751	96.2
CA	0.812	0.558	0.884	0.74	88.5
CBAM	0.821	0.575	0.879	0.758	84
SE	0.828	0.566	0.87	0.759	94.3

#### Improvement of YOLOv5s and ablation experiments

After the attention mechanism is selected, this paper continues to analyze and compare the detection results. To address the issue of occlusion in the results, Soft-NMS is employed for enhancement, while the utilization of the BiFPN network aims to augment the model’s feature extraction capability. Therefore, the CIoU Loss is improved to Focal-EIoU Loss in the model, the improved method is added to the YOLOv5s network, and the ablation experiment is carried out, and the outcomes are displayed in Table 4.

**Table 4:** The improvement of YOLOv5s and ablation results.

Algorithm	mAP (IoU = 0.5)	mAP (IoU = 0.5:0.95)	P	R	FPS
YOLOv5s	0.819	0.573	0.87	0.751	96.2
YOLOv5s + SE	0.828	0.566	0.87	0.759	94.3
YOLOv5s + BiFPN	0.823	0.565	0.86	0.76	85.5
YOLOv5s + EIoU	0.815	0.568	0.884	0.734	86.2
YOLOv5s + Soft-NMS	0.746	0.539	0.902	0.701	70.4
YOLOv5s + SE + EIoU	0.801	0.547	0.828	0.748	84
YOLOv5s + SE + Soft-NMS	0.754	0.535	0.879	0.716	73.5
YOLOv5s + BiFPN + Soft-NMS + EIoU + SE	0.749	0.531	0.882	0.714	68
YOLOv5s + BiFPN + Soft-NMS	0.753	0.545	0.897	0.719	63.7
BiFEL-YOLOv5s	0.828	0.577	0.865	0.779	83.8

**Figure 9:**
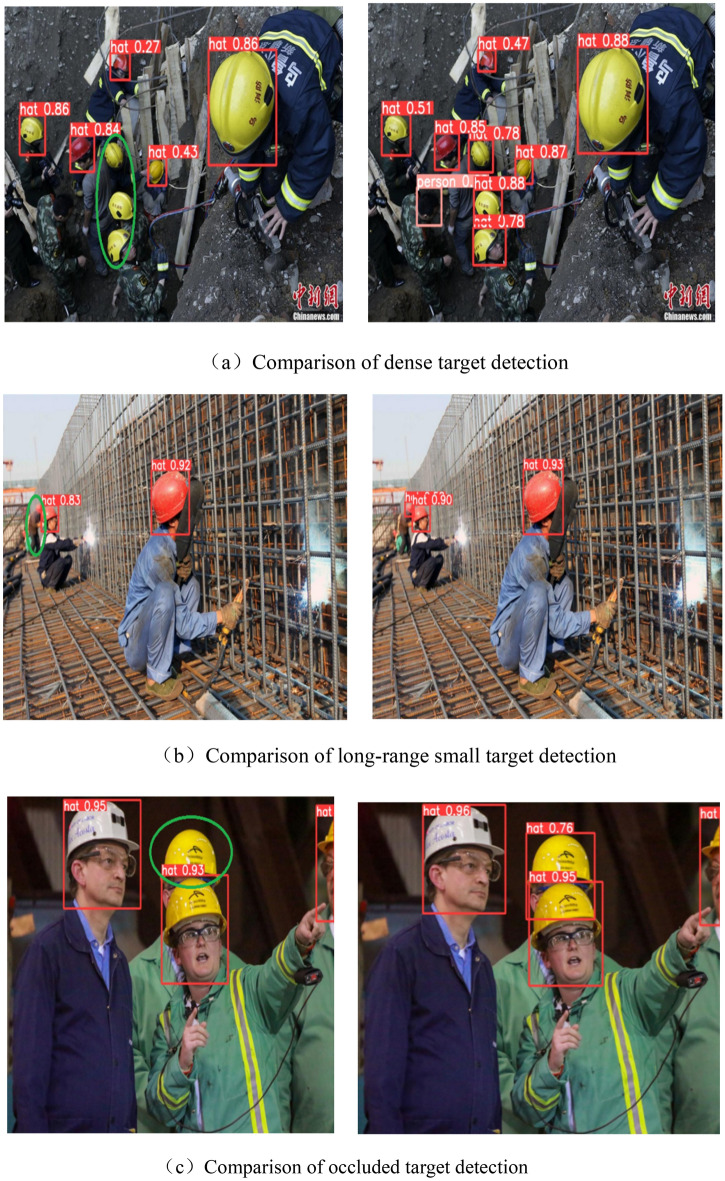
Comparison of network detection results between BiFEL-YOLOv5s and original YOLOv5s in different scenarios respectively.

From the experimental results, among the four improved methods, SE attention mechanism and BiFPN network will improve the average accuracy of the network, Focal-EIoU Loss and Soft-NMS will improve the accuracy of the network. Therefore, in this case, this paper combines the two methods of improvement direction in pairs. For safety helmet detection, with a view to ensure the safety of construction workers, the recall rate of the model should be guaranteed to be high enough to detect as many people as possible who do not wear safety helmets. From the comparison results, the method of improving BiFPN in Neck based on YOLOv5s and combining Focal-EIoU Loss has a better effect on safety helmet detection, with an average precision increase of 0.9% and a recall rate increase of 2.8%, and the detection speed of the model does not decrease too much. We have christened it “BiFEL-YOLOv5s” as a novel approach for safety helmet object detection, and Fig. 9 illustrates the comparison of its detection outcomes.

**Figure 10:**
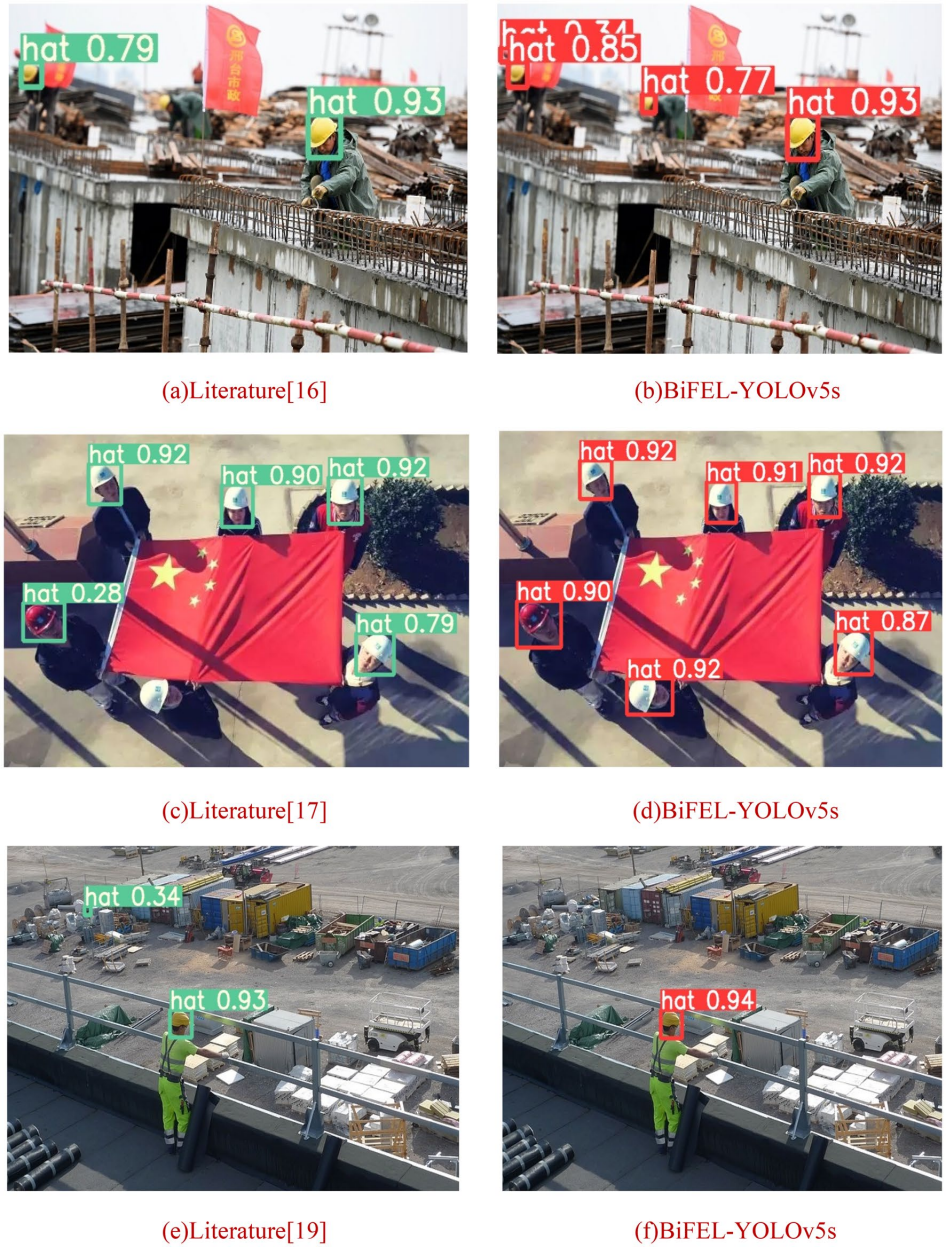
Comparison results with major helmet improvement algorithms.

Since there is no public dataset in the literature^16–20^, and each literature uses different datasets to get different results, in order to ensure the validity and intuition of the comparison results, a unified dataset is now used, i.e., the SHWD dataset used in this paper. The comparison results are shown in Table 5 and Fig. 10.

**Table 5:** Table of comparison results with major helmet improvement algorithms.

Attention mechanism	mAP (IoU = 0.5)	mAP (IoU = 0.5:0.95)	P	R	FPS
Improvement of Faster RCNN^16^	0.803	0.544	0.822	0.792	12.9
Improvement of YOLOv4^19^	0.794	0.441	0.884	0.74	34.28
Improvement of YOLOv5^17^	0.812	0.526	0.853	0.752	110
BiFEL-YOLOv5s	0.828	0.577	0.865	0.779	83.8

From the Table 5 and Fig. 10, it can be seen that the detection accuracy of literature^16^ is higher, but due to the two-stage target detection algorithm, the detection speed is low, and there is a serious leakage of small target samples; Literature^17^ has a higher detection speed, but there is a certain degree of leakage; Literature^19^ has a moderate detection accuracy and speed, but there is a misdetection. Comprehensive comparison, this paper's proposed detection algorithm has the highest accuracy, and the detection speed is lower than that of Literature^17^, at the same time there is a significant improvement in the leakage of small targets and the misdetection of the situation.

## Conclusion

This paper takes the safety helmet object in the construction environment as the research object and studies the safety helmet object detection method based on deep learning. Firstly, this paper analyzes the significance of real-time detection of safety helmet objects, understands the related object detection methods, and determines that YOLO algorithm is more suitable for safety helmet object detection. The running environment of Python and Pytorch is built, different versions of YOLO network are used to train it, and the experimental results are compared and analyzed. Considering the long training time of YOLOv7 and the high requirements for hardware equipment, YOLOv5s is selected as the basic network. Aiming at the improvement of YOLOv5s basic network, considering its shortcomings for small objects and occluded objects, SE, CBAM, and CA attention mechanisms are added to the backbone network respectively. By analyzing and comparing experimental outcomes, it is concluded that the channel attention mechanism, SE, is better suited for safety helmet object detection. Focal-EIoU is employed to enhance the loss function, while Soft-NMS is utilized to enhance post-processing non-maximum suppression, thus improving both model accuracy and detection speed. The final experimental results show that our proposed BiFEL-YOLOv5s method has a better effect on safety helmet object detection, and can meet the real-time object detection in complex work scenes.

## Data Availability

The datasets used and/or analysed during the current study available from the corresponding author on reasonable request.
